# Walking to work: The role of walkability around the workplace in a Dutch adult commuting population

**DOI:** 10.1016/j.ssmph.2023.101578

**Published:** 2023-12-10

**Authors:** Tea Osmënaj, Thao Minh Lam, Alfred J. Wagtendonk, Nicolette R. den Braver

**Affiliations:** a Amsterdam UMC location Vrije Universiteit Amsterdam, Epidemiology and Data Science, De Boelelaan 1117, Amsterdam, the Netherlands; b Amsterdam Public Health Institute, Health Behaviours and Chronic Diseases, Amsterdam, the Netherlands; c Upstream Team, Vrije Universiteit, Amsterdam, the Netherlands; d The National Institute for Public Health and the Environment (RIVM), Bilthoven, the Netherlands

**Keywords:** Physical activity, Residential walkability, Workplace walkability, Commute walking

## Abstract

Current evidence on neighborhood walkability and active commuting focuses on residential rather than workplace environment. This cross-sectional study investigated whether higher workplace walkability (WW) was associated with commute walking, both independently and together with residential walkability, using data from 6769 respondents of the 2017 Dutch national travel survey. In a fully adjusted logistic regression model, 10% increase in WW was associated with 32% higher odds of commute walking (Odds ratio (OR): 1.31, 95% Confidence Interval (CI: 1.27–1.36). The estimates were stronger in rural dwellers than urban residents, (OR_rural_ 1.49, 95%CI: 1.34–1.64 vs OR_highly_._urban_ 1.19, 95%CI: 1.13–1.26). In participants with both high residential walkability and WW, we observed 215% higher odds (OR 3.15, 95% CI: 2.48–3.99) of commute walking compared to those with low walkability in both. Our study indicated the importance and complementary nature of walkable residence and workplace in contribution to physical activity of working individuals through active commuting.

## Highlights

• Walkability around the workplace is associated with commute walking.
• This association was stronger for those living in rural areas.
• Residential and workplace walkability are complementary for commute walking.
• Above associations are consistent when all-purpose walking was used as outcome.

## 1 Introduction

Physical inactivity is one of the most prominent public health issues with about 2 million attributable deaths every year (World Health Organization, 2020). Of the adult population worldwide, 23% fail to adhere to the World Health Organization's (WHO) recommended guidelines for physical activity on health (van der Ploeg & Bull, 2020). Physical inactivity results in low energy expenditure, which can lead to weight gain and negative health implications, including abnormal glucose metabolism, cardiometabolic morbidity and increased all-cause mortality (Dooris et al., 1998; Panahi & Tremblay, 2018; World Health Organization, 2018).

Physical inactivity has increased over the years, mainly because occupations have become more sedentary (Knuth & Hallal, 2009) and commuting to work is mostly (77%) done by passive means of transport (e.g., driving) (Brownson, Boehmer, & Luke, 2005; Herman & Larouche, 2021). People spend an average of five to 9 h sitting per day, while 10 h of sitting is associated with 34% higher risk of all-cause mortality (Chau et al., 2013). Incorporating physical activity in work-related activities is associated with better cardiometabolic health and cardiorespiratory fitness in active commuters (Henriques-Neto et al., 2020). Early evidence showed that commute walking (defined as walking to/from places of work or study) was linked to 21–27% reduction in cardiovascular risk and mortality (Dinu, Pagliai, Macchi, & Sofi, 2019) improved physical fitness in adults of all genders (Barengo et al., 2004; Matthews et al., 2007), as well as significantly reduced risk of all-cause mortality (Dinu et al., 2019). Moreover, a study with the UK Biobank indicated that switching from driving to more active mode of commute was associated with 0.30 kg/m^2^ reduction of BMI over a median of 4.4 years of follow-up (Flint, Webb, & Cummins, 2016). Population-level physical activity (PA) intervention strategies aiming at commute walking can address public health challenges.

According to socio-ecological models, a variety of factors both personal (e.g., family composition, occupation, overall health) and environmental (e.g. neighborhood walkability) can affect PA behaviors such as walking (Booth, 2001; Amarasinghe & D’Souza, 2012). According to Geoffrey Rose's prevention paradox, addressing these environmental determinants might have small impact on individuals but larger impact at population level; as opposed to individual-level interventions which may lead to significant changes in a small number of high-risk individuals (Rose, 2001). Therefore, targeting such environmental level determinants could potentially deliver far-reaching public health benefits.

Neighborhood walkability is a composite measure of built environment characteristics that facilitate walking, including higher population density, street connectivity and land use diversity (Forsyth, 2015). Neighborhood walkability has been shown to be associated with higher total PA in an international study across 12 countries worldwide (Sallis et al., 2016) and lower risk of type 2 diabetes (Den Braver et al., 2018). However, most studies on walkability focus almost exclusively on residential neighborhoods, which are substantially different from workplace neighborhoods (Howell et al., 2017; Hurvitz & Moudon, 2012). The latter, however, could potentially be an important determinant for modal choice with subsequent consequences on health. For instance, a recent study conducted in Canada (Den Braver, 2022) suggested that drivability around the workplace neighborhood is a stronger determinant of car use than drivability around residential neighborhood, making the workplace an interesting target for intervention.

To date, only a handful of studies investigated objective workplace walkability and walking behaviors. An Australian study (Barr et al., 2019) found that working at the most walkable areas (defined as having 10 to 12 types of destinations within an 800m street network buffer) was associated with 8.7 extra minutes of walking for any purposes, compared to working at least walkable areas (<4 destination types). Marquet et al. (2020) found positive associations between workplace walkability (measured with either WalkScore© or a land use-based index) and accelerometer-based PA in female employees in the USA, both in PA while at work and PA within the proximity of work. Lin et al. (2022) found that lower workplace walkability (measured with WalkScore©) was associated with more time spent sitting in cars and less time sitting in public transport in desk-working adults in Japan. Though limited, these results suggest possible contribution of workplace walkability to commute walking and PA in general. Studies investigating subjective measures of walkability found that convenient walking routes, with maintained pavements and efficient public transportation around the workplace increase the odds of commute walking in the UK (Adams, Bull, & Foster, 2016). Work neighborhoods perceived to have higher land use mix or higher street connectivity or safer for pedestrians and traffic was also associated with more active trips in 32 neighborhoods around Seattle and Baltimore areas (Carlson et al., 2018). Finally, greater objectively-measured commute distance from home to work was associated with lower active commute, while self-reported free or low-cost recreation facilities around the workplace were positively associated with active commute (Yang et al., 2014). However, self-reported walkability components could not be combined, replicated, or validated, reducing comparability with other studies.

Even fewer studies were able to capture the combined effect of residential and workplace walkability on walking. Carlson et al. (2018) examined perceived home and workplace walkability and found that adding workplace walkability components on top of residential walkability increased the total explained variance for objectively measured total moderate-to-vigorous PA, transport-related PA and self-reported active commute in adults in Baltimore and Seattle. Two other cross-sectional studies assessed objectively measured combined walkability both found that workplace walkability was a stronger determinant of self-reported walking in Australian adults (Barr et al., 2019) and active transport (including biking) in Canadian college students (Howell et al., 2017) than the home walkability. Overall, these three studies suggested walkability at workplace and at home have independent association with transport-related PA, hence it is important to consider them both in our study. However, there is little use of formal high-quality walkability indices, domain-specific physical activity outcomes (commute walking, total physical activity), and only very limited evidence for the combined effect workplace and residential walkability, especially in European context.

Therefore, in this study we aimed to investigate the association between objectively-measured walkability around the workplace and commute walking in the Dutch population, using a comprehensive and validated walkability index (Lam et al., 2021). Additionally, we aimed to investigate the association between combined workplace and residential neighborhood walkability and walking for commute. We hypothesized that high workplace walkability is associated with more walking, and based on previous research, association is probably stronger for those living in rural areas.

## 2 Methods

### 2.1 Study setting

We used data from the 2017 Dutch National Travel Survey (in Dutch: Onderzoek Verplaatsingen in Nederland (OViN); Statistics Netherlands, 2018). The aim of OViN is to provide information about the daily mobility of the Dutch population. The survey contained data on trip level e.g., duration and mode, and individual level e.g., demographic and household characteristics. In this study, a nationwide validated walkability index was linked to the 4-digit postal codes of participants’ residence and workplace. In the Netherlands, 4-digit postal code (PC) areas are geographically delineated areas within municipalities and include approximately 1870 households and have an average area size of 3.1 km^2^ (Timmermans et al., 2018). The nationwide walkability index was developed with spatial data from the Geoscience and Health Cohort Consortium (Lakerveld et al., 2020).

### 2.2 Study population

A representative sample of 38,127 individuals participated in the 2017 version of OViN. For the purpose of this study, we included only the working population, thus excluding 15,972 individuals younger than 18 and older than 65 years old (not in working age) and excluding those who did not work nor follow education (n = 4800). We excluded individuals who did not leave the house on the day of the survey, for reasons such as sickness and individuals who travelled abroad because walkability could not be determined outside the Netherlands (n = 2828). Then, we excluded based on exposure factors. Individuals whose work address and residential address were identical were excluded because we hypothesized that travel distance would be the main determinant of transport mode choice, and that residential and workplace walkability index would overlap which would limit us from distinguishing between walkability at destination and trip origin. Further, subjects who had missing work or residential PC were excluded, because walkability could not be determined. In total 7388 individuals were excluded based on the exposure criteria. Lastly, individuals who had incomplete information of covariates of interest were also excluded (n = 370). This resulted in an analytic sample of 6769 participants (Table 1).

**Table 1:** Study population exclusion steps, based on chosen criteria.

Step	N excluded	N remained
Full sample	–	38,127
**Work status:** - Outside working age - Unemployed or not studying	20,772	17,355
**Outcome related:** - Stayed indoors during the survey - Went abroad	2828	14,527
**Exposure related:** - Same home & work postal code - Missing home or work postal code	7388	7139
**Missing covariates:**	370	6769
**Analytic sample**		**6769**

### 2.3 Neighborhood walkability

Neighborhood walkability was measured using a validated, nationwide walkability index. The development of the walkability index was described in detail by Lam et al. (2022). In short, the index consisted of seven different components: population density, density of retail and service destinations, land use mix, street connectivity, green space, sidewalk density and density of public transport stops. The number of residents per hectare was used to measure population density. Land use mix was calculated using an entropy index, which evaluated how equally land use classes were spread. Land use classes were as identified by the National Georegister, i.e., commercial; socio-cultural services; residential areas; offices and public services; green space and recreation. Street connectivity was defined as the density of real intersections along pedestrian-accessible road segments. Green space density included the proportion of land covered by parks, public gardens, forests, and cemeteries. Sidewalk density was defined as the proportion of sidewalks in a given area. Public transport density was the point density of all railway stations, trams, buses, metros, and ferries.

For each of the seven components, we rasterized raw data into 25m × 25m cells and performed focal statistics for each of the 3 circular buffer sizes (150m, 500m and 1000m) and aggregated them to PC4 level (n = 4068). Each walkability component was z-standardized, and the walkability index was calculated as an average across all componential z-scores. The overall walkability score was scaled to range between 0 and 100, with a higher value indicating a more walkable neighborhood. The 150-m Euclidian buffer size was used for the main analyses since it was found that to be most relevant for walking in the Dutch context (Lam et al., 2022). The other buffer areas (500m and 1000m) were used for sensitivity analysis.

For the primary research question, we linked this walkability index (both continuous and in quintiles) to participants’ workplace PC4 address. For the second research question, we created a variable to combine residential and workplace walkability. Using the median as a cut-off point, we categorized residential and workplace walkability each into high- and low-walkable. These categories were then combined into one variable with four categories (in the order of home and workplace walkability): low/low; high/low; low/high; high/high.

### 2.4 Outcome assessment

Data from OViN (Onderzoek Verplaatsingen in Nederland (OViN); Statistics Netherlands, 2018) for 2017 were used to estimate walking behaviors for a representative sample of the Dutch population. Briefly, respondents were asked to complete a travel diary for one particular day including information on trip origin, destinations, purposes, modes of transportation and travel length for each trip. For this study, we selected trips with the purpose to travel ‘to/from work’ and ‘to/from school’ to capture all commuting trips. We then aggregated the minutes of walking for each commuting trip to the total number of minutes walked for commute per person on the survey day. For sensitivity analyses, we calculated total number of minutes walked per person per day regardless of trip purpose, to assess the broader impact of workplace walkability and combined walkability on total walking.

Since minutes of walking were highly zero-inflated in both commute and total walking, we dichotomized these variables into walkers (people who walked at least 1 min for commute or in total) versus non-walkers (people who did not walk for commute or at all on survey day).

### 2.5 Covariates

The OViN survey captured self-reported individual and household characteristics of participants. Age was divided in three classes (18–34, 35–50, 50–65 years old); sex in male or female; and social participation was categorized as full-time work, part-time work, or student. Day of the survey was determined by study design and was dichotomized to weekdays and weekend. Highest attained education was categorized into low (no education/primary education), medium (secondary education) and high education (tertiary education). Household composition was categorized into living alone, living with partner, living with family and others (e.g., living with housemate). Number of cars in household was categorized into no cars, one car, and more than one car. Urbanization degree was categorized into highly urban (>2500 addresses/km^2^), urban (1000–2500 addresses/km^2^) and rural (<1000 addresses/km^2^). Lastly, the standardized annual dispensable household income was categorized into low (<30.000 euro), medium (30.000–40.000 euro) or high (>40.000 euro) accordingly to the 2016 Dutch household income tertiles (Statistics Netherlands, 2016).

### 2.6 Statistical analysis

Descriptive statistics were presented for the total analytical sample and in quintiles of workplace walkability. For continuous variables, mean ± standard deviation was presented for normally distributed variables and otherwise in median [interquartile range (IQR)]. Counts and percentages (N, %) were presented for all categorical variables.

Logistic regression was performed to analyze firstly the association between workplace walkability at 150m buffer size (both continuous and in quintiles) and odds of walking for commute. Effect estimates were presented as ORs with 95% CI. We presented a p-for-trend over the quintiles to assess the linearity of the association, where a p-value <0.05 indicates linearity. Besides the crude model, two *a priori* adjusted models were presented: model 1 adjusted for demographic characteristics: sex, age classes, social participation, education level, household composition, standardized outcome, model 2 additionally adjusted for travel and survey related characteristics, namely number of cars per household and day of survey. Nagelkerke R^2^ was calculated as a measure of model fit, in each model of the main analysis. We also investigated effect modification by age groups, sex, income, workplace urbanization degree and residential urbanization degree by including interaction terms in the model, and associations were stratified in case of a significant interaction (p-value <0.05).

Secondly, logistic regression was performed to analyze the association between combined walkability index (in four categories) at 150m buffer size and odds of walking for commute without and with adjusting for confounders (respectively crude, model 1 and model 2 similarly to main analyses). We tested for the same effect modifiers in this secondary analyses, and stratified analyses if appropriate.

For sensitivity analyses, we additionally investigated odds of total walking as an outcome to assess the impact of workplace and combined walkability on total walking. Furthermore, we also examined if associations remained consistent across walkability indices measured at 500m and 1000m Euclidian buffer sizes. All analyses were conducted in SPSS version 26.0 BMI (2017)

## 3 Results

### 3.1 Descriptive statistics

Table 2 summarizes the baseline characteristics of the total study population and in quintiles of workplace walkability index. Of the 6769 included participants, 55.1 % were male, had a mean age of 41.3 (SD = 13.3) years old, were highly educated (42.6%), lived with a partner (57.1%) in an urban area (between 1000 and 2500 addresses/km^2^, 42.7%). In contrast, those who worked in areas of highest quintile of walkability were slightly younger (mean age 40.5, SD = 13.0) but also highly educated (54.2%), lived with a partner (53.7%) and worked in highly urban areas (more than 2500 addresses/km^2^, 92.3%). Of the total sample, 15% walked for commute and they walked a median of 18.0 [IQR = 20.0] minutes on the survey day; compared to 31.7% for those in the highest quintile of walkability, but duration was similar at 18.0 min [IQR = 18.0]. In general, 26% of the sample walked for any purpose on survey date, and they walked a median of 99.0 [IQR = 67.0] minutes; in the highest quintile these numbers were 42.6% and 95.0 [IQR = 67.0] respectively.

**Table 2:** Baseline characteristics for participants of OVIN 2017 in total and across quintiles of workplace walkability, presented in n (%) or mean ± SD or median [IQR].

	Total N = 6769	Quintile 1 N = 1371	Quintile 2 N = 1281	Quintile 3 N = 1312	Quintile 4 N = 1398	Quintile 5 N = 1407
**Sex (male)**	3728 (55.1%)	873 (63.7%)	760 (59.3%)	719 (54.8%)	673 (48.1%)	703 (50.0%)
**Age (mean±SD)**	41.3 ± 13.3	41.9 ± 13.3	42.0 ± 13.2	41.1 ± 12.9	41.2 ± 14.1	40.5 ± 13.0
18-34	2294 (33.9%)	408 (29.8%)	394 (30.8%)	453 (34.5%)	510 (36.5%)	529 (37.6%)
35-50	2494 (36.9%)	541 (39.5%)	493 (38.5%)	474 (36.1%)	479 (34.3%)	507 (36.0%)
51-65	1981 (29.3)	422 (30.8%)	394 (30.8%)	385 (29.3%)	409 (29.3%)	371 (26.4%)
**Household composition**						
Live alone	1109 (16.4%)	226 (16.5%)	194 (15.1%)	197 (15.0%)	216 (15.5%)	276 (19.6%)
Couple	3861 (57.1%)	769 (56.1%)	756 (59.0%)	759 (57.9%)	821 (58.7%)	756 (53.7%)
Parents with children	1799 (26.6%)	376 (27.4%)	331 (25.8%)	356 (27.1%)	361 (25.8%)	375 (26.7%)
**Education level**						
Low	993 (14.7%)	257 (18.7%)	218 (17.0%)	207 (15.8%)	163 (11.7%)	148 (10.5%)
Medium	2896 (42.8%)	655 (47.8%)	573 (44.7%)	570 (43.4%)	601 (43.0%)	497 (35.3%)
High	2880 (42.6%)	459 (33.5%)	490 (38.3%)	535 (40.8%)	634 (45.4%)	762 (54.2%)
**Cars per household**						
No car	624 (9.2%)	86 (6.3%)	64 (5.0%)	74 (5.6%)	159 (11.4%)	241 (17.1%)
One car	3031 (44.8%)	548 (40%)	547 (42.7%)	573 (43.7%)	640 (45.8%)	723 (51.4%)
More than one	3114 (46.0%)	737 (53.7%)	670 (52.3%)	665 (50.7%)	599 (42.8%)	443 (31.5%)
**Weekdays**						
weekend	482 (7.1%)	104 (7.6%)	91 (7.1%)	91 (6.9%)	89 (6.4%)	107 (7.6%)
weekday	6287 (92.9%)	1267 (92.4%)	1190 (92.9%)	1221 (93.1%)	1309 (93.6%)	1300 (92.4%)
**Workplace urbanization degree**						
Highly urban (>2500 addresses/km^2^)	1946 (28.7%)	0 (0.0%)	19 (1.5%)	74 (5.6%)	562 (40.0%)	1291 (92.3%)
Urban (1000–2500 ad/km^2^)	2890 (42.7%)	150 (10.9%)	669 (52.2%)	1122 (85.4%)	841 (59.9%)	108 (7.7%)
Rural (<1000 ad/km^2^)	1933 (28.6%)	1221 (89.1%)	593 (46.3%)	118 (9.0%)	1 (0.1%)	0 (0.0%)
**Standardized income**						
Low (<30.000 €)	2962 (43.8%)	647 (47.2%)	555 (43.3%)	602 (45.9%)	589 (42.1%)	569 (40.4%)
Middle (30–40.000 €)	2071 (30.6%)	428 (31.2%)	421 (32.9%)	401 (30.6%)	412 (29.5%)	409 (29.1%)
High (>40.000 €)	1736 (25.7%)	296 (21.6%)	305 (23.8%)	309 (23.6%)	397 (28.4%)	429 (30.5%)
**Occupation**						
Part-time	1380 (20.4%)	232 (16.9%)	265 (20.7%)	316 (24.1%)	318 (22.7%)	249 (17.7%)
Full-time	4786 (70.6%)	1081 (78.8%)	943 (73.6%)	899 (68.5%)	887 (63.4%)	976 (69.4%)
Student	603 (9.0%)	58 (4.2%)	73 (5.7%)	97 (7.4%)	193 (13.8%)	182 (12.9%)
**People who actively commuted (N, %)**	1009 (14.9%)	92 (6.7%)	114 (8.9%)	122 (9.3%)	235 (16.8%)	446 (31.7%)
Minutes of commute walking, median [IQR]	18.0 [20.0]	12.0 [17.0]	18.0 [25.0]	15.5 [24.0]	20.0 [20.0]	18.0 [18.0]
**People who walked at all (N, %)**	1781 (26.3%)	263 (19.1%)	250 (19.5%)	270 (20.5%)	398 (28.5%)	600 (42.6%)
Total minutes walked, median [IQR]	99.0 [67.0]	94.0 [61.0]	102.0 [65.0]	99.0 [72.0]	105.0 [71.0]	95.0 [67.0]

### 3.2 Workplace walkability and commute walking

In a fully adjusted model, each 10% increase (corresponding to 10-point increment) in workplace walkability at 150m buffer size was associated with 32% higher odds of walking for commute (OR: 1.32, 95% CI: 1.27–1.36; p_trend_: <0.001). In categorical exposure models, the highest quintile workplace walkability was associated with 345% higher odds of walking for commute, compared to the lowest walkability quintile (Q5 vs Q1 OR: 4.45, 95% CI: 3.47–5.73) (Table 3).

**Table 3:** Logistic regression for the associations between (1) quintiles of workplace walkability index at 150m buffer size or (2) continuous workplace walkability (per 10-point increment) and odds of walking for commute. The odds ratios and 95% confidence intervals are presented. N = 6769.

Models	Categorical workplace walkability						Continuous workplace walkability
	Q1	Q2	Q3	Q4	Q5	P for trend	
**Crude**	ref	**1.36** (1.02; 1.81)	**1.43** (1.08; 1.89)	**2.79** (2.17; 3.60)	**6.47** (5.09; 8.23)	<0.001	**1.40** (1.36; 1.45)
Nagelkerke's R^2^: **0.11**							**0.11**
**Model 1**	ref	1.32 (0.99; 1.76)	1.29 (0.98; 1.72)	**2.23** (1.72; 2.90)	**5.44** (4.25; 6.97)	<0.001	**1.37** (1.32; 1.42)
Nagelkerke's R^2^: **0.17**							**0.17**
**Model 2**	ref	1.32 (0.98; 1.77)	1.28 (0.95; 1.70)	**1.94** (1.49; 2.54)	**4.45** (3.47; 5.73)	<0.001	**1.32** (1.27; 1.36)
	Nagelkerke's R^2^: **0.21**						**0.21**

Model 1: Adjusted for demographics: sex, age, education level, social participation, household composition, std income.

Model 2: Additionally adjusted for number of cars per household, weekdays.

The associations between workplace walkability and walking for commute were modified by residential urbanization degrees (p for interaction: <0.001, Table 4); but not by workplace urbanization degrees, participant age, sex or income. For people living in rural areas, each 10% increase in workplace walkability was associated with 49% higher odds of walking for commute in a fully adjusted model (OR: 1.49, 95% CI: 1.34–1.64; p_trend_: <0.001), compared to 19% in highly urban areas (OR: 1.19, 95% CI: 1.13–1.26; p_trend_: <0.001) and 33% in urban areas (OR: 1.33, 95% CI: 1.26–1.40; p_trend_: <0.001) (Table 4).

**Table 4:** Logistic regression for the association between continuous workplace walkability index at 150m buffer size (per 10-point increment) and odds of walking for commute, stratified by residential urbanization degrees (highly urban, urban, rural). The odds ratios and 95% confidence intervals are presented. N = 6769.

Strata (N_walked_/N_total_)	Continuous workplace walkability	Nagelkerke's R2
**Highly urban** (541/1597)		
Model 1	**1.21** (1.15; 1.28)	**0.09**
Model 2	**1.19** (1.13; 1.26)	**0.13**
**Urban** (350/3275)		
Model 1	**1.37** (1.30; 1.44)	**0.17**
Model 2	**1.33** (1.26; 1.40)	**0.22**
**Rural** (118/1897)		
Model 1	**1.51** (1.37; 1.66)	**0.23**
Model 2	**1.49** (1.34; 1.64)	**0.26**
**All strata** (1009/6769)		
Model 1	**1.37** (1.32; 1.42)	**0.17**
Model 2	**1.32** (1.27; 1.36)	**0.21**

Model 1: Adjusted for demographics: sex, age, social participation, education level, household composition, standardized income.

Model 2: Additionally adjusted for number of cars per household, weekdays.

### 3.3 Combined residential and workplace walkability

In the investigation of combined walkability, when both residential and workplace walkability were high, association with commute walking was highest (OR: 3.15, 95% CI: 2.48–3.99) (Table 5). Even when either home or workplace walkability was high, odds of walking was still significantly higher compared to those living in low walkability for both (respective OR: 1.99 (95% CI: 1.52–2.61) and 2.81 (95% CI: 2.18–3.62). There was no significant effect modification by age, sex, income or urbanization degree, either residential or workplace.

**Table 5:** Logistic regression for the association between combined walkability index (residential/workplace) at 150m buffer size and odds of walking for commute. The odds ratios and 95% confidence intervals are presented. N = 6769.

Residential/Workplace walkability (N_walked_/N_total_)	Category 1 Low/Low (101/1895)	Category 2 High/Low (162/1279)	Category 3 Low/High (229/1378)	Category 4 High/High (517/2217)
**Crude**	1	**2.58** (1.99; 3.34)	**3.54** (2.77; 4.53)	**5.40** (4.32; 6.75)
Nagelkerke's R^2^: **0.07**				
**Model 1**	1	**2.46** (1.89; 3.21)	**2.99** (2.33; 3.84)	**4.39** (3.49; 5.52)
Nagelkerke's R^2^: **0.14**				
**Model 2**	1	**1.99** (1.52; 2.61)	**2.81** (2.18; 3.62)	**3.15** (2.48; 3.99)
Nagelkerke's R^2^: **0.18**				

Model 1: Adjusted for demographics: sex, age, education level, social participation, household composition, std income.

Model 2: Additionally adjusted for number of cars per household, weekdays.

### 3.4 Sensitivity analyses

For total walking, in a fully adjusted model, each 10% increase in workplace walkability at 150m buffer size was associated with 13% higher odds of total walking (OR: 1.13, 95% CI: 1.07–1.19; p_trend_: <0.001). In categorical exposure models, the highest quintile of workplace walkability was associated with 52% higher odds of walking, compared to the lowest quintile (Q5 vs Q1 OR: 1.52, 95% CI: 1.11–2.07) (Table S1). For combined walkability, when both residential and workplace walkability were high, association with total walking was highest (OR: 1.69, 95% CI: 1.40–2.04) (Table S2). Even when only home or workplace walkability was high, odds of walking was still significantly higher compared to those living in low walkability for both (respective OR: 1.25 (95% CI: 1.02–1.53) and 1.49 (95% CI: 1.25–1.78). Compared to analysis using commute walking as outcome, association with total walking showed some attenuation though significance largely remained.

When workplace walkability was measured at larger buffer sizes (500m, 1000m), associations with commute walking were also slightly attenuated (Supplementary Tables S3 and S4 versus Table 2), but the overall direction of association and significance remained consistent across different buffer sizes.

## 4 Discussion

In this study, we showed that every 10% increase in workplace walkability was associated with 32% higher odds of walking for commute, which were strongest in residents of rural areas (49%). High combined workplace and home neighborhood walkability was most strongly associated with commute walking. The same patterns of association were observed with total walking as an outcome, although associations were attenuated.

Our primary results were in line with other studies conducted on objectively measured walkability at workplace in other parts of the world, including Howell et al. (2017), Barr et al. (2019) and Marquet et al. (2020) who found significant association between workplace walkability and total walking or objectively measured-PA. Our study strengthened these findings by using a more precise outcome, namely commute walking. Nevertheless, associations held when investigating total walking as outcome. This illustrated the impact that targeting workplace walkability may have on daily PA levels. Moreover, to our knowledge, there was no previous evidence for differences between urban and rural dwellers. Our stratified results showed a stronger association between workplace walkability and odds of commute walking for people living in rural areas. This result was in line with Yang et al. (2014) where longer distance to workplace (i.e. potentially living more rural areas) was associated with less active commuting. This illustrated that destination walkability could potentially play a more important role for rural residents (who were more inclined to drive to work anyway) compared to their urban counterparts. This also mirrored the finding in our earlier study on residential walkability, where urbanization degree was a significant effect modifier with strongest associations observed in rural residents (Lam et al., 2022).

As secondary analysis, we assessed the association of combined walkability with walking for commute and observed that when both residential and workplace walkability were high, the odds of walking for commute was highest; followed by high work/low residential and low work/high residential combinations. This was in line with earlier studies by Howell et al. (2017) and Barr et al. (2019) where walkability at various locations were compared in terms of their association with total active transport (walking or biking). Higher workplace and residential walkability were associated with 8.3 (95% CI: 7.3–9.3) and 3.9 (95% CI: 2.3–5.5) minutes of walking respectively (Howell et al., 2017). In comparative terms, both studies suggested that study- or workplace walkability was more predictive of (total) walking than residential walkability which was also suggested by our results. Our sensitivity analysis further showed that combined walkability was more relevant for commute walking, even though associations with total walking was still significant. Interestingly, Howell et al. (2017) found that averaging walkability across the full physical activity space resulted in the strongest associations with odds of active transport, suggesting that beyond nondiscretionary locations such as home and work, walkability of other daily locations also play a role. Future studies of walkability in the Netherlands could potentially take this into account to reduce the issue of uncertain geographic context problem (Kwan, 2012).

The strengths of the present study were first the use of a comprehensive, high-resolution and validated walkability index (Lam et al., 2022). Second, different buffer sizes for the index were used in analysis, improving the confidence in consistency of these findings independent of areal units. Third, OViN is a very extensive travel survey, which provides a wealth of information on a geographically representative sample of the Dutch population. In particular, having extensive data on trip level including starting and ending points allowed for precise exposure and outcome assessments of walkability and walking. Moreover, due to the large sample size, we were able to perform stratified analyses, providing insight into the role of demographic and contextual variables.

However, some limitations from our study should also be addressed. First, this was a cross-sectional study, so we could not infer causal relations between walkability and walking. Second, all walking outcomes were self-reported, so responses may have been more prone to recall bias as opposed to objective measures such as accelerometer-based PA. Furthermore, one study showed that OViN respondents were shown to underreport short trips, cycling and walking trips, and non-home-based excursions when compared to a place-based survey (Hoogendoorn-Lanser, Schaap, & OldeKalter, 2015). Lastly, the results may be limited to the Netherlands, since built environmental features may differ significantly from those in other countries. As a result, this walkability index will require validation and evaluation when applied to other countries or regions.

## 5 Implications for practice

Our results showed that working in more walkable environment was associated with higher odds of active commuting. Increasing walkability levels around workplaces can therefore contribute to increasing physical activity of employees through commuting. Public health policymakers and urban planners could benefit from this study by utilizing the walkability index score to assess the overall quality of work neighborhood. After identifying targeted neighborhoods, urban planners could choose to focus on specific components of the index, for example public transport or green space, to improve the walkability of these neighborhoods. While more and more policy initiatives target neighborhood walkability in general (POLIS Network, 2021), this study adds an extra perspective on the workplace where people also spend a significant proportion of their day. Moreover, we argue that workplace and residential walkability have synergistic effects on walking, suggesting that both should be taken into consideration during infrastructure planning (*The Living Environment Information Point (IPLO)*, 2023). This highlights a potential target for intervention to improve PA and subsequently, overall health and well-being.

## 6 Conclusion

In conclusion, our study showed that a higher workplace walkability was associated with higher odds of walking for commute, both independently and in combination with residential walkability. We emphasized the importance and complementary nature of walkable residential and workplace in contribution to physical activity of working individuals through active commuting.

## Data statement

Walkability data are available upon requests via GECCO website (www.gecco.nl). Transport survey (OViN 2017) data are publicly available and can be downloaded free of charge via following website (in Dutch): https://easy.dans.knaw.nl/ui/datasets/id/easy-dataset:103498.

## Funding

The Geoscience and Health cohort consortium (GECCO) was financially supported by the Netherlands Organization for Scientific Research (NWO), the Netherlands Organization for Health Research and Development (ZonMw) and Amsterdam University Medical Centers. More information on GECCO can be found at the following link: www.gecco.nl. Thao M. Lam and Nicolette R. den Braver received funding from the Netherlands Organization for Scientific Research (NWO) under the Exposome-NL consortium (NWO-Gravitation Grant number: 024.004.017). Alfred J. Wagtendonk received funding from the Netherlands Organization for Health Research and Development (ZonMw) to collect and process environmental data used in this study. The other author declared no conflict of interest.

## Ethical statement

There is no ethical statement for this study because the study made use of existing data, which were also anonymised. The travel survery data used were publicly available.

## Data Availability

Data will be made available on request.
