# Supplementary material for: Walking to work: The role of walkability around the workplace in a Dutch adult commuting population
Source: SSM Popul Health. 2023 Dec 10;25:101578. doi: 10.1016/j.ssmph.2023.101578 (PMC10761905; doi:10.1016/j.ssmph.2023.101578)
Supplement: Multimedia component 1 [file mmc1.docx]

# **Walking to work: The role of walkability around the workplace**

**Supplemental materials**

**Table S1.** Odds ratios and 95% confidence intervals for the association of workplace walkability index quintiles to total minutes walked per day

| Models | Categorical workplace walkability | | | | | | Continuous workplace walkability |
| --- | --- | --- | --- | --- | --- | --- | --- |
|  | Q1  (263/1371) | Q2  (250/1281) | Q3  (270/1314) | Q4  (398/1404) | Q5  (600/1399) | P for trend |  |
| Crude | ref | 1.02  (0.84;1.24) | 1.09  (.09;1.32) | **1.67**  (1.39;1.99) | **3.16**  (2.67;3.75) | <0.001 | **1.24**  (1.21;1.28) |
| Model 2 | ref | 0.86  (0.69;1.07) | **0**.**77**  (0.59;0.99) | 0.91  (0.69;1.19) | **1.52**  (1.11;2.07) | <0.001 | **1.13**  (1.07;1.19) |

Model 2: fully adjusted

**Table S2.** Odds ratios and 95% confidence intervals for the association of combined walkability index categories to total minutes walked per day

| Residential / Workplace walkability  (N_walked_/ N_total_) | Category 1  Low / Low  (319/1895) | Category 2  Low / High  (369/1378) | Category 3  High / Low  (311/1279) | Category 4  High / High  (782/2217) |
| --- | --- | --- | --- | --- |
| Crude | 1 | **1.81**  (1.53;2.14) | **1.58**  (1.33;1.89) | **2.69**  (2.32;3.12) |
| Model 2 | 1 | **1.49**  (1.25;1.78) | **1.25**  (1.02;1.53) | **1.69**  (1.40;2.04) |

Model 2: fully adjusted

**Table S3.** Odds ratios and 95% confidence intervals for the association of workplace walkability index to active commuting. Buffer size 1000 meters

| Models | Categorical workplace walkability | | | | | | Continuous workplace walkability |
| --- | --- | --- | --- | --- | --- | --- | --- |
|  | Q1  (92/1371) | Q2  (114/1281) | Q3  (122/1312) | Q4  (235/1398) | Q5  (446/1407) | P for trend |  |
| Crude | ref | **1.44**  (1.11;1.86) | **1.79**  (1.39; 2.30) | **3.07**  (2.43;3.87) | **6.87**  (5.51;8.57) | <0.001 | **1.40**  (1.36;1.44) |
| Model 2 | ref | 1.32  (0.97; 1.79) | 1.41  (0.95;2.10) | **1.96**  (1.30;2.94) | **3.79**  (2.41;5.95) | <0.001 | **1.25**  (1.18;1.33) |

Model 2: fully adjusted

**Table S4.** Odds ratios and 95% confidence intervals for the association of active commuting to workplace walkability index. Buffer size 500 meters

| Models | Categorical workplace walkability | | | | | | Continuous workplace walkability |
| --- | --- | --- | --- | --- | --- | --- | --- |
|  | Q1  (92/1371) | Q2  (114/1281) | Q3  (122/1312) | Q4  (235/1398) | Q5  (446/1407) | P for trend |  |
| Crude | ref | **1.32** (1.03;1.71) | **1.78**  (1.39; 2.28) | **2.93**  (2.33;3.69) | **6.41**  (5.15;7.97) | <0.001 | **1.44**  (1.39;1.48) |
| Model 2 | ref | 1.21  (0.90; 1.63) | 1.39  (0.97;2.01) | **1.75**  (1.19;255) | **2.32**  (2.11;4.81) | <0.001 | **1.29**  (1.22;1.38) |

Model 2: fully adjusted
